# Environmentally sustainable biogenic fabrication of AuNP decorated-graphitic g-C_3_N_4_ nanostructures towards improved photoelectrochemical performances†

**DOI:** 10.1039/c8ra00690c

**Published:** 2018-04-16

**Authors:** Mohammad Ehtisham Khan, Mohammad Mansoob Khan, Moo Hwan Cho

**Affiliations:** School of Chemical Engineering, Yeungnam University Gyeongsan Gyeongbuk 38541 South Korea mhcho@ynu.ac.kr mehtishamkhan1@gmail.com +82-53-810-4631 +82-53-810-2517,; Chemical Sciences, Faculty of Science, Universiti Brunei Darussalam Jalan Tungku Link Gadong BE1410 Brunei Darussalam mmansoobkhan@yahoo.com

## Abstract

Noble-metal gold (Au) nanoparticles (NPs) anchored/decorated on polymeric graphitic carbon nitride (g-C_3_N_4_), as a nanostructure, was fabricated by a simple, single step, and an environmentally friendly synthesis approach using single-strain-developed biofilm as a reducing tool. The well deposited/anchored AuNPs on the sheet-like structure of g-C_3_N_4_ exhibited high photoelectrochemical performance under visible-light irradiation. The Au-g-C_3_N_4_ nanostructures behaved as a plasmonic material. The nanostructures were analyzed using standard characterization techniques. The effect of AuNPs deposition on the photoelectrochemical performance of the Au-g-C_3_N_4_ nanostructures was examined by linear sweep voltammetry (LSV), electrochemical impedance spectroscopy (EIS), incident photon-to-current efficiency (IPCE) and cyclic voltammetry (CV) in the dark and under visible-light irradiation. The optimal charge transfer resistance for Au-g-C_3_N_4_ nanostructures (6 mM) recorded at 18.21 ± 1.00 Ω cm^−2^ and high electron transfer efficiency, as determined by EIS. The improved photoelectrochemical performance of the Au-g-C_3_N_4_ nanostructures was attributed to the synergistic effects between the conduction band minimum of g-C_3_N_4_ and the plasmonic band of AuNPs, including high optical absorption, uniform distribution, and nanoscale particle size. This simple, biogenic approach opens up new ways of producing photoactive Au-g-C_3_N_4_ nanostructures for potential practical applications, such as visible light-induced photonic materials for real device development.

## Abstract 2

Proposed schematic model for the biogenic synthesis of Au-*g*-C_3_N_4_ nanostructures using an environment-friendly approach.

## Graphical abstract

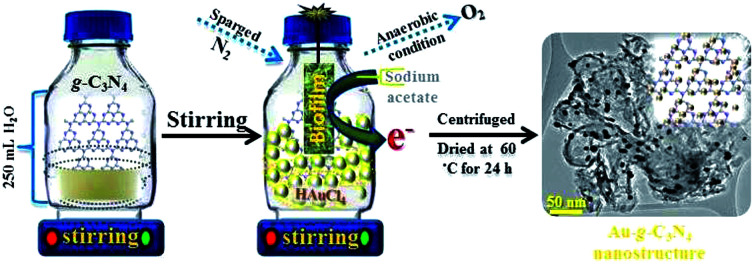

## 1. Introduction

Green chemistry focuses mainly on the reduction, recycling or removal of toxic and hazardous chemicals in various fabrication processes by finding creative, alternative routes for producing the desired products with a less adverse impact on the environment and human health. Green chemistry is a more eco-friendly green alternative to conventional chemistry practices.^1^ The development of environmentally friendly methodologies in material synthesis is of great importance to expand their visible light-induced applications in electrochemical analysis.^2,3^ Currently, noteworthy research efforts have been devoted to the realization of efficient, economical, and green sources for the fabrication of nanoparticles with a well-defined chemical composition, size, and morphology for applications in many cutting-edge technological areas.^4–7^ Single strain developed biofilms is one of the positive hopes for the fabrication of carbon-based metal nanostructures.^8^ In general, biofilms form on solid surfaces by different kinds of micro-organism for their mutual benefits. Here, a biofilm was developed using a single strain *Shewanella oneidensis*, which is an electrochemically active microorganism that can be used to control reactions in a range of fields, such as chemical/biological synthesis and bioremediation.^8,9^ Nanoparticles of noble metals, such as Au, Ag, Pt, and Pd can strongly absorb visible light from the solar spectrum^10,11^ owing to their special effect of surface plasmon resonance (SPR), which can be adjusted by varying their size and shape.^12–15^ The size and shape-dependent optical and electronic properties of metal nanoparticles have made them attractive for interfacial charge transfer in semiconductor–metal nanostructures.^14,16,17^ Plasmonic AuNPs work as a visible light absorber and a thermal redox active center.^18–21^ Considering the advantages of AuNPs, it is probable that the photoelectrochemical performance of g-C_3_N_4_ can be improved further after the successful anchoring of AuNPs.^22^ Polymeric graphitic carbon nitride (g-C_3_N_4_) with a band gap of 2.7 eV and long range π–π conjugation is a stable allotrope with a stacked two-dimensional structure under ambient conditions.^23–25^ Compared to its inorganic semiconductor counterparts, g-C_3_N_4_ is a sustainable and environmentally friendly organic semiconductor material that consists of carbon and nitrogen, which are among the most abundant elements on Earth. Since Wang *et al.* first reported that novel molecular photo-based material g-C_3_N_4_ nanostructures exhibited photoactivity for H_2_ production, considerable efforts have been made to synthesize g-C_3_N_4_ through the heat treatment of numerous nitrogen-rich organic precursors.^26,27^ Metal-free π-conjugated g-C_3_N_4_ nanostructures have interesting electronic properties as well as high thermal and chemical stability, making them valuable materials for visible light-driven electrochemical analysis.^26,28–30^

In the present study, a novel, simple and biogenic/green synthesis approach was applied for the fabrication of Au-g-C_3_N_4_ nanostructures. The successful anchoring of AuNPs onto the sheet-like structure of g-C_3_N_4_ was optimized using HAuCl_4_ precursor (1 mM, 3 mM, and 6 mM), and it was found that anchoring with up to 6 mM of AuNPs resulted in improved photoelectrochemical performance. The effects of small amounts of AuNPs (1 mM, 3 mM, and 6 mM) anchored successively onto sheet-like structures of g-C_3_N_4_ to improve the visible-light absorption performance and separate the photogenerated electron–hole pairs were studied. The as-fabricated nanostructures exhibited improved photocurrent performance under the visible-light irradiation. The photoelectrochemical performance was tested based on the SPR effects of AuNPs, lower band gap energy, low photoluminescence intensity, excellent visible-light absorption, and superior photocurrent generation. The charge transfer properties in the Au-g-C_3_N_4_ nanostructures highlight its potential as good quality plasmonic-based electronic material for energy storage and conversion applications for real device fabrication.

## 2. Experimental section

### 2.1. Materials

Hydrogen tetrachloroaurate(iii) hydrate (HAuCl_4_·*n*H_2_O; *n* = 3.7) from Kojima Chemicals, Japan. Urea (98.0%), ethyl cellulose, and α-terpineol (C_10_H_18_O) were acquired from KANTO Chemical Co., Japan. Sodium acetate (CH_3_COONa) and sodium sulfate (Na_2_SO_4_) were obtained from Duksan Pure Chemicals Co. Ltd., South Korea. Fluorine-doped transparent conducting oxide glass (FTO; F-doped SnO_2_ glass; 7 Ω sq^−1^) was acquired from Pilkington, USA. The bacterial culture medium was purchased from Becton Dickinson and Company (NJ, USA). All other chemicals were of analytical grade and used as received. The solutions were prepared from DI water obtained using a PURE ROUP 30 water purification system.

### 2.2. Methods

X-Ray diffraction (XRD, PANalytical, X'pert-PRO MPD) was performed using Cu Kα radiation (*λ* = 0.15405 nm). The diffuse absorbance/reflectance ultraviolet-visible spectra (DRS) of the powder pure g-C_3_N_4_ and Au-g-C_3_N_4_ nanostructures samples were obtained using an ultraviolet-visible-near infrared (UV-VIS-NIR) double beam spectrophotometer (VARIAN, Cary 5000, USA) equipped with a diffuse reflectance accessory. A given amount of the g-C_3_N_4_ and Au-g-C_3_N_4_ nanostructure powder was pressed uniformly in the sample holder, which was then placed at the integrating sphere for the absorbance/reflectance measurements. The photoluminescence (PL, Kimon, 1 K, Japan) of the samples was recorded over the scanning range, 300–800 nm, using an excitation wavelength of 325 nm. The BET specific surface area of the samples was measured using a Belsorp II-mini (BEL, Japan Inc.). The microstructure was examined by field emission transmission electron microscopy (FE-TEM, Tecnai G2 F20, FEI, USA) operating at an accelerating voltage of 200 kV. Selected-area electron diffraction (SAED) and high angle annular dark field (HAADF) observations were carried out on the same transmission electron microscope. Quantitative analysis was performed by energy dispersive spectrometry (EDS) attached to the transmission electron microscope. X-ray photoelectron spectroscopy (XPS, ESCALAB 250 XPS System, Thermo Fisher Scientific U.K.) was conducted using the following X-ray source: monochromated Al Kα radiation, *hν* = 1486.6 eV; X-ray energy, 15 kV; 150 W; spot size, 500 μm; take-off angle, 90; pass energy, 20 eV; BE resolution, 0.6 eV (calibrated using Ag 3d_5/2_) at the Center for Research Facilities, Yeungnam University, South Korea. XPS fitting was done using “AVANTAGE” software by a Shirley subtraction and the shape of the peaks used for the deconvolution was Gaussian–Lorentzian shapes. The sensitivity factor used for Au 3d_5_ was 30.5.

Photoelectrochemical analyses, such as linear sweep voltammetry (LSV), electrochemical impedance spectroscopy (EIS), and cyclic voltammetry (CV), were performed using a potentiostat (Versa STAT 3, Princeton Research, USA) comprised of a standard three-electrode system. Ag/AgCl (3 M KCl), a Pt gauge, and FTO glass coated with the pure g-C_3_N_4_ and Au-g-C_3_N_4_ nanostructures were used as the reference, counter and working photoelectrode, respectively. The experiment LSV and EIS were performed in a 0.2 M sodium sulphate (Na_2_SO_4_) solution as the supporting electrolyte at room temperature and CV was performed in a 0.2 M phosphate buffer solution (pH 7; 0.2% PBS). The projection area of the photoelectrode was 1 cm^2^. The working electrodes were prepared as follows: 100 mg of each sample was mixed thoroughly by adding ethyl cellulose as a binder and α-terpineol as the solvent. The mixture was stirred and heated on a hot plate with a magnetic stirrer until a thick paste was obtained. The paste obtained was then coated on a FTO glass substrate using the doctor-blade method and kept drying overnight under a 60 W lamp; the electrode was later used as a photoelectrode for the photoelectrochemical measurements.

### 2.3. Development and fabrication of single strain biofilm on carbon foam

Carbon foam was prepared using a melamine sponge (Dae Han Co. Ltd, Korea) as the template.^31^ The biofilm on the carbon foam was prepared using the procedure reported elsewhere.^21,32^ Carbon foam (without wet proof, Fuel Cell Earth LLC) with a size of 2.5 × 4.5 cm^2^ was used as an anode electrode instead of carbon paper. In the anode chamber, Luria Broth (LB) medium was inoculated with overnight cultures of *Shewanella oneidensis* at a ratio of 1 : 100. The LB media was purged with N_2_ gas for 10 min to remove the environmental oxygen and maintain the anaerobic conditions. The fully developed biofilm on the carbon foam was confirmed using a microbial fuel cell by obtaining the appropriate voltage. The living biofilm formed on the carbon foam specimens was used to synthesize the series of Au-g-C_3_N_4_ nanostructures.

### 2.4. Single strain developed biofilm synthesis of Au-g-C_3_N_4_ nanostructures (1 mM, 3 mM and 6 mM)

**Scheme 1:**
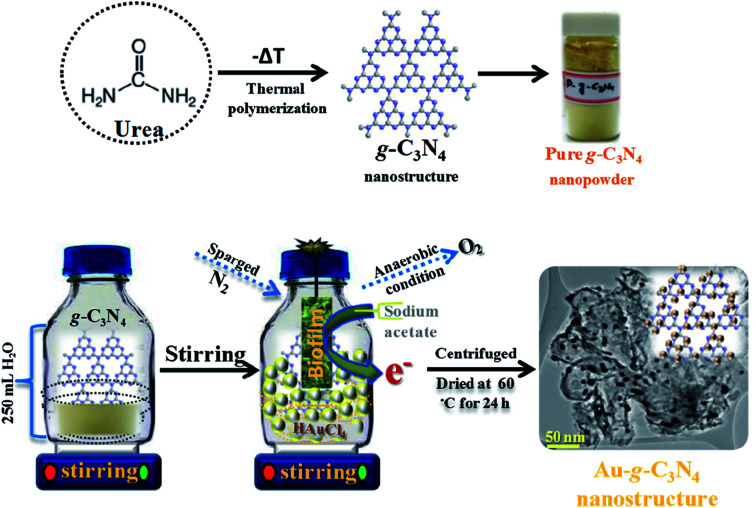
Projected schematic model for the biogenic synthesis of the Au-g-C_3_N_4_ nanostructures using an environmentally friendly approach.

Graphitic g-C_3_N_4_ was prepared using a facile single pot method by the modest heating of urea at 550 °C in a muffle furnace for 4 h with a ramping rate of 20 °C min^−1^ under air flow conditions. The resulting material was then naturally cooled to room temperature, the whitish yellow color powder was extract as a sheet-like structure of pure g-C_3_N_4_ (ref. 33 and 34) (Scheme 1).

Three setup arrangements of 200 mL of aqueous suspensions of pure g-C_3_N_4_ and 1 mM, 3 mM, and 6 mM Au^3+^ were prepared. The mixture of pure g-C_3_N_4_ and HAuCl_4_ (Au^3+^ ions) was stirred for 15 min to allow the adsorption of Au^3+^ ions onto the sheet-like g-C_3_N_4_ structure. Subsequently, the optimal amount of sodium acetate (0.2 g) was added individually to the suspension as an electron contributor. The reaction mixtures were sparged with nitrogen (N_2_) gas for 5 min to sustain an anaerobic environment. The single strain developed biofilm were hung individually in a reaction bottle and the setup was sealed and left for magnetic stirring at 30 °C. The reaction mixture setups were stirred for a further 6 h to complete the reaction. In each case, the initial white color changed to a dark pink color within 30 min, which was the sign of the reduction of Au^3+^ to Au^0^. Finally, purple to light purple precipitates were obtained in the 1 mM, 3 mM, and 6 mM HAuCl_4_ cases, respectively. The reaction mixtures were centrifuged and the powdered Au-g-C_3_N_4_ nanostructures were isolated for further characterization and photoelectrochemical studies.

Two precise syntheses were performed to examine the role of the single strain developed biofilm and sodium acetate. Two 5 mM g-C_3_N_4_ aqueous suspensions (200 mL) were prepared. In the first controlled synthesis, an aqueous solution containing a 0.2 g sodium acetate and 1 mM HAuCl_4_ was added. In the second controlled synthesis, only a 3 mM HAuCl_4_ aqueous solution was added. Both reaction mixtures were sparged with N_2_ gas for 5 min to sustain the anaerobic environment. The developed biofilm were hung in the second controlled synthesis only. Both systems were sealed and stirred with a magnetic stirrer at 30 °C. No variations were detected, even after 48 h. These long-established reaction steps confirmed that the biofilm and sodium acetate are essential to complete the synthesis of the Au-g-C_3_N_4_ nanostructures.

### 2.5. Photoelectrochemical studies of pure g-C_3_N_4_ and Au-g-C_3_N_4_ nanostructures as a photoelectrode performance

The photoelectrochemical performance of the pure g-C_3_N_4_ and Au-g-C_3_N_4_ nanostructures was examined by LSV, EIS and CV under ambient conditions in the dark and under visible light irradiation. The LSV and EIS experiments were performed in 50 mL of an aqueous 0.2 M Na_2_SO_4_ solution in the dark and under visible light irradiation at room temperature. The photocurrent response was examined by LSV in the dark and under visible light irradiation at a scan rate of 50 mV s^−1^ over the applied potential range, −1.0 to 1.0 V. EIS which were conducted using a 400 W lamp under visible light irradiating intensity of 31.0 mW cm^2^ (3M, USA) with frequencies ranging from 1 to 10^4^ Hz at 0.0 V *vs.* Ag/AgCl in potentiostatic mode. The CV experiments were conducted in 50 mL of 0.1 M PBS (phosphate buffer solution) in the dark and under visible light irradiation. CV analysis was performed at a scan rate of 50 mV s^−1^. The incident photon-to-current efficiency (IPCE), used to investigate the photoresponsivity of nanostructures using the xenon lamp with specific wavelength filters to select the required wavelength of light.

## 3. Results and discussion

### 3.1. Standard characterization of pure g-C_3_N_4_ and Au-g-C_3_N_4_ nanostructures

#### 3.1.1. Structural, purity and phase confirmation analysis of pure g-C_3_N_4_ and Au-g-C_3_N_4_ nanostructures

**Fig. 1:**
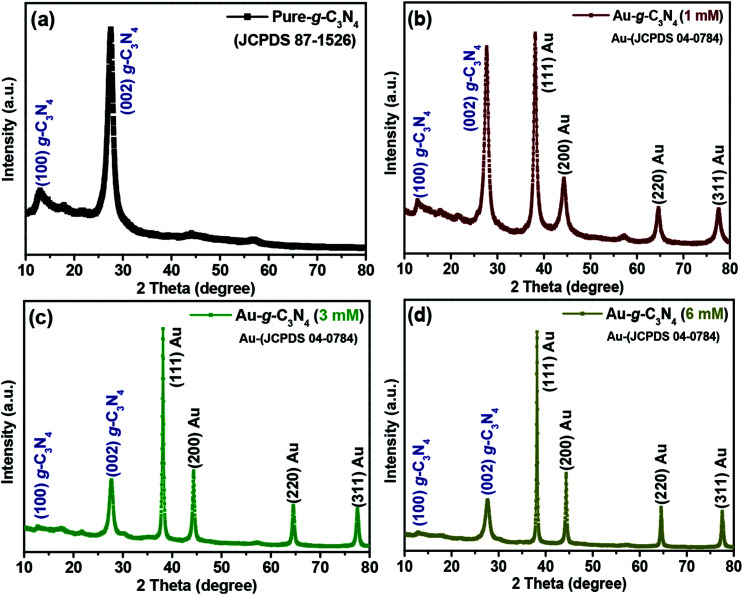
Representative XRD patterns of (a) pure g-C_3_N_4_, (b, c and d) 1 mM, 3 mM and 6 mM of Au-g-C_3_N_4_ nanostructures.

X-Ray diffraction was performed to explore the crystal structure, phase, and purity of pure g-C_3_N_4_, as shown in Fig. 1(a). The XRD pattern of pure g-C_3_N_4_ showed two peaks at 13.1° and 27.3° 2*θ*. The small peak at 13.1° 2*θ* was assigned to the (100) plane with *d* = 0.676 nm and the other strong peak at 27.3° 2*θ* corresponded to *d* = 0.324 nm due to the long-range interplanar stacking of the aromatic arrangement and it is recognized as the (002) plane of pure g-C_3_N_4_ (JCPDSD 87-1526).^35^ Additionally, the two additional weak diffractions peaks appeared at ∼43° and ∼58° which can be attributed to the planes of graphitic carbon nitride. This outcome from the denser packing or a distortion of the mesopores structure in which every second the arrangement of mesopores sheet is displaced.^36^ Notably, the mentioned peaks were disappeared in case of 3 mM and 6 mM while small peak appeared in case 1 mM because there was small concentration of Au ions.

In the case of (1 mM, 3 mM and 6 mM) of AuNPs-decorated g-C_3_N_4_ samples, the XRD patterns revealed four separate reflections at 38.1° (111), 44.4° (200), 64.8° (220) and 77.6° (311) for the AuNPs, in addition to the peaks for g-C_3_N_4_. The observed reflections were well matched with the AuNPs in the prepared nanostructures corresponding to the reported JCPDS values (04-0784).^37^ The intensity of the peaks for the AuNPs increased gradually with increasing loading of Au^3+^ ions onto the sheet-like structure of g-C_3_N_4_. The four peaks confirmed the anchoring of AuNPs onto the g-C_3_N_4_ surface, which was clearly absent in the pure g-C_3_N_4_ sample; no other extra/impurity peaks were found in the as-fabricated samples. The presence of both Au planes and g-C_3_N_4_ confirmed the successful formation of the Au-g-C_3_N_4_ nanostructures using the green/biogenic synthesis approach.

The mean crystallite size of the g-C_3_N_4_ and Au-g-C_3_N_4_ nanostructures were calculated using the Scherrer's formula,

1 *D* = *κλ*/*β* cos *θ*

where *κ* is the shape factor and has a typical value of ∼0.9, *λ* is the wavelength (Cu Kα = 0.15405 nm), *β* is the full width at half maximum of the most intense peak (in radians), and *θ* is the main peak of g-C_3_N_4_, which was observed at 27.43° 2*θ*. The calculated crystallite size of bare g-C_3_N_4_ from the most intense peak at 27.43° 2*θ* was 6.6 nm and the calculated crystallite size of the Au-g-C_3_N_4_ nanostructures from the most intense peak was 12.2, 22.9, and 27.9 nm, respectively. This shows that the crystallite size of the Au-g-C_3_N_4_ nanostructures increased because of the anchoring of AuNPs on to the g-C_3_N_4_ sheets compared to pure g-C_3_N_4_. These increased crystallite values further confirmed the successful fabrication of the Au-g-C_3_N_4_ nanostructures.

#### 3.1.2. Optical and photoluminescence analysis of pure g-C_3_N_4_ and Au-g-C_3_N_4_ nanostructures

**Fig. 2:**
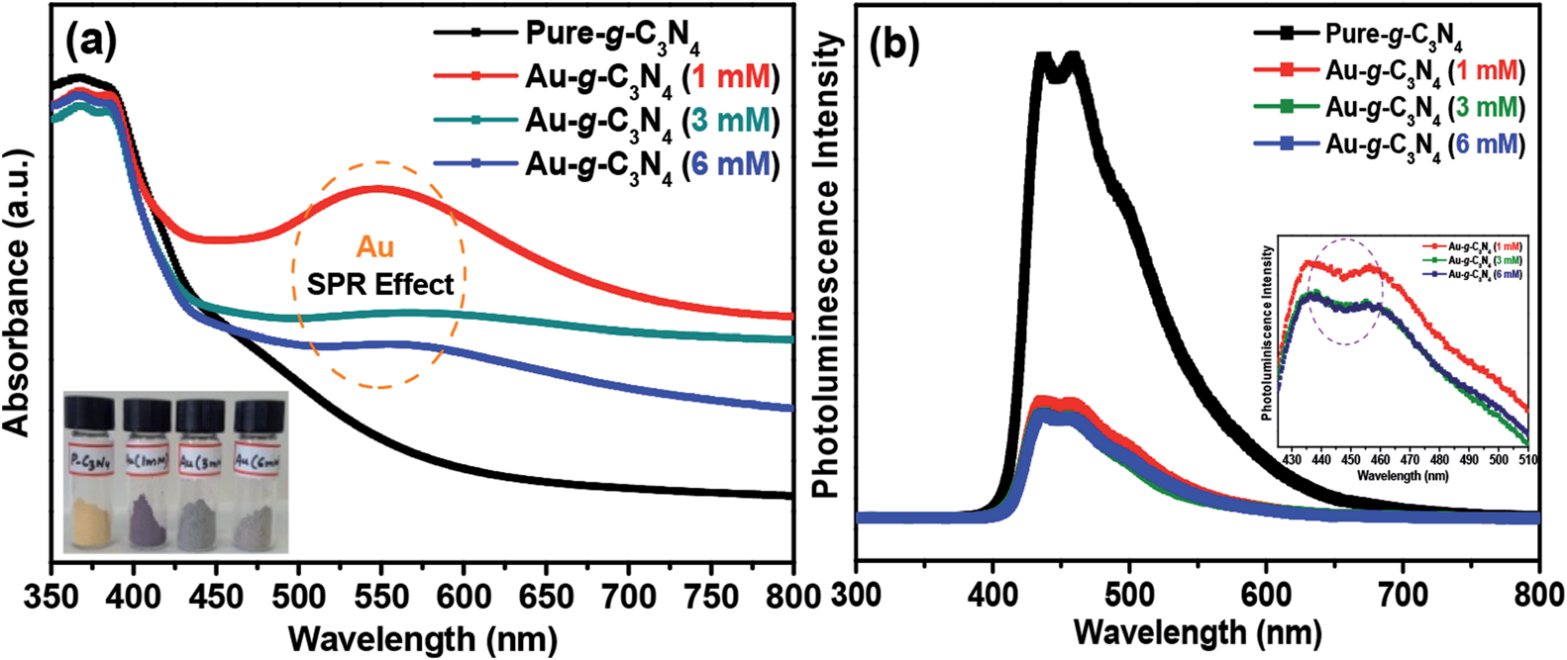
(a) UV-Vis absorbance spectra, and (b) photoluminescence spectra of pure g-C_3_N_4_ and Au-g-C_3_N_4_ nanostructures.

Fig. 2(a and b) shows the optical absorbance and photoluminescence analysis of the pure g-C_3_N_4_ and Au-g-C_3_N_4_ nanostructures. The present spectrum showed a high absorbance value in the range, 475–525 nm, because of the SPR band characteristics of the AuNPs, which showed that the AuNPs had been anchored successfully onto the g-C_3_N_4_ samples and showed the improved visible light absorption of AuNPs.^38,39^ In addition to the confinement effect, the interparticle coupling contributes to the SPR broadening of AuNPs decorated g-C_3_N_4_ nanostructures as well. It is due to the particle interactions which increase in local field fluctuations, giving rise to an extensive range of photon energies for plasmon resonance to take place.^17^ From the absorbance spectra in Fig. 2(a), there was a red shift in the absorbance band of the Au-g-C_3_N_4_ nanostructures compared to that of pure g-C_3_N_4_. The inset in Fig. 2(a) shows that the AuNPs decorated onto g-C_3_N_4_, in the 1 mM sample displayed a purple color, which was a clear indication of the successful reduction of Au^3+^ to Au^0^ and the fabrication of AuNPs. Fig. S1† presents the typical reflectance spectra from 360–780 nm wavelengths, showing improved reflectance in the case of the Au-g-C_3_N_4_ nanostructures, which further confirmed the successful formation of Au-g-C_3_N_4_ nanostructures.

Fig. 2(b) shows the photoluminescence (PL) spectra of the pure g-C_3_N_4_ and Au-g-C_3_N_4_ nanostructures. These spectra are very helpful for examining the migration, transfer of charge carriers, and separation and recombination processes of the photogenerated electron–hole pairs.^13,18^ The PL intensity is exceedingly reliant on the electron–hole pair recombination processes. The PL intensity is dependent on electron–hole pair recombination processes. The as-fabricated pure g-C_3_N_4_ and Au-g-C_3_N_4_ nanostructures materials showed only one type of PL intensity in the recorded spectra. The broad luminescence peak at 455 nm was assigned to the band–band PL phenomenon with a light energy approximately equal to the band gap energy of the g-C_3_N_4_ and Au-g-C_3_N_4_ nanostructures for the photoelectrode performance.^13,40^ As the PL intensity is inversely related to the charge recombination between the photogenerated electron–hole pairs, the anchoring/decoration of the AuNPs onto the sheet-like structure of g-C_3_N_4_ could prevent charge recombination between the opposite charge carriers, leading to improved photoelectrochemical performance.^40,41^ The overall PL studies of the Au-g-C_3_N_4_ nanostructures clearly showed higher charge transfer ability, which could be responsible for the improved photoelectrochemical performance. On the other hand, the inset in Fig. 2(b) shows that there is no shift in the emission wavelength of 455 nm. In addition, two emission centers were observed in the shorter excitation wavelength (436.0 nm and 458.8 nm) which was in contrast to that observed for longer excitation wavelengths. The PL intensity decreased gradually with increasing wavelength as the corresponding excitation energy is reduced in the case of (1 mM to 6 mM) of Au-g-C_3_N_4_ nanostructures.

#### 3.1.3. High resolution transmission electron microscopy (HR-TEM) image of pure g-C_3_N_4_ and Au-g-C_3_N_4_ nanostructures

**Fig. 3:**
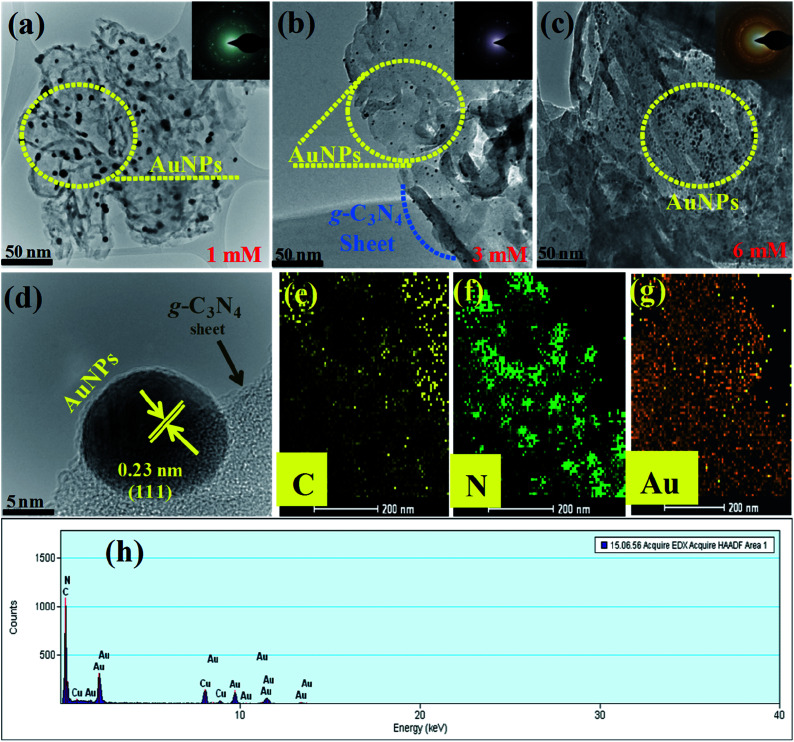
(a, b and c) TEM images of the 1 mM, 3 mM, and 6 mM of Au-g-C_3_N_4_ nanostructures shows the uniform presence of AuNPs onto g-C_3_N_4_ (the inset shows the SAED crystal ring pattern); (d) HR-TEM image showing the interface between the AuNPs and g-C_3_N_4_, lattice fringes of AuNPs onto the g-C_3_N_4_; (e, f and g), showing elemental mapping of C (yellow color), N (green color), and Au (metallic gold color); (h) shows the elemental composition of the Au-g-C_3_N_4_ nanostructures.

The surface structures, morphology, and particle size of the as-fabricated samples Au-g-C_3_N_4_ nanostructures were investigated by TEM and HR-TEM. As shown in Fig. 3(a), the particles with a dark color can be assigned to AuNPs and the sheet like gray color area was assigned to the sheet-like structure of g-C_3_N_4_. The as-synthesized Au-g-C_3_N_4_ nanostructures displayed a sheet like morphology with a few layered structures. The sheets consisted mainly of graphitic planes with a conjugated aromatic system.^13^ The flat surface of the g-C_3_N_4_ sheet acts as a visible-light absorber. The TEM images (a, b and c) show that the number of AuNPs increases with increasing concentration of the Au precursor. The SAED pattern of the nanostructure showed a series of bright concentric rings, suggesting that the as-fabricated sample is polycrystalline in nature (inset of Fig. 3(a–c)).

The mean diameter of the AuNPs was in the range, 12–15 nm, and the nanoparticles were clearly attached to the surface and edges of the g-C_3_N_4_ sheet. Fig. 3(d) clearly showed the interfacial interaction of AuNPs with the sheet-like structure of g-C_3_N_4_, which also covered the intact surface area of the sheet uniformly. The lattice fringes of the Au 0.23 nm (111) plane for metallic Au indicated the crystalline behavior of the samples, which further confirmed the presence of the AuNPs and the good interaction at the interface of the g-C_3_N_4_ sheet. The elemental mapping presented in Fig. 3(e, f and g) shows C (yellow), N (green), and Au (metallic gold), which provides strong evidence for the existence of carbon, nitrogen, and AuNPs anchored successfully onto the sheet-like structure of g-C_3_N_4_. Fig. 3(h) shows the elemental composition of the Au-g-C_3_N_4_ nanostructures without any other elemental peak. Fig. S2(a–d)† presents HR-TEM images of the Au-g-C_3_N_4_ nanostructures. The average particle size distribution graph of Fig. S3(a–c)† screening the average particle size is ranging between 12–15 nm.

#### 3.1.4. XPS of pure g-C_3_N_4_ and Au-g-C_3_N_4_ nanostructures

**Fig. 4:**
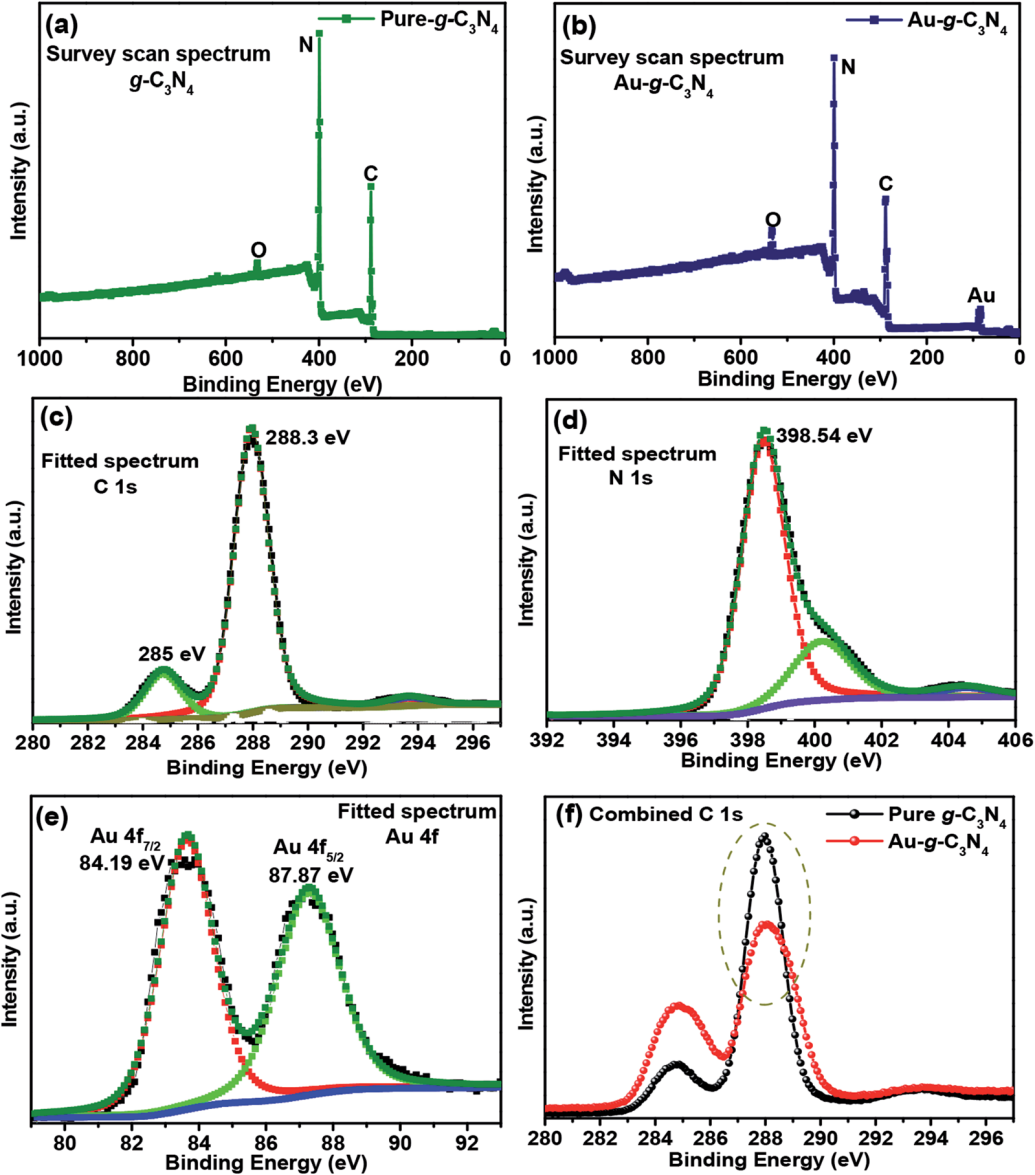
(a and b) XPS survey scan spectra of pure g-C_3_N_4_ and Au-g-C_3_N_4_ nanostructures, (c, d and e) fitted spectra of C 1s, N 1s and Au 4f, and, (f) combined spectra of pure g-C_3_N_4_ and Au-g-C_3_N_4_ nanostructures.

XPS is a surface-specific characterization tool that can be used to confirm the chemical environment and elemental oxidation state. XPS was carried out on the pure g-C_3_N_4_ and Au-g-C_3_N_4_ nanostructures in the region, 0–1000 eV (Fig. 4). Consequently, XPS was used to determine the formal oxidation state of all the elements present in the pure g-C_3_N_4_ and Au-g-C_3_N_4_ (1 mM, 3 mM, and 6 mM). Fig. 4(a) displayed the elemental composition of pure g-C_3_N_4_, in which two major peaks were assigned to C and N and a small peak for oxygen at ∼531 eV, which might be some hydroxyl groups (–OH) attached to the surface of g-C_3_N_4_. No impurity peak was observed.^42^ The survey scan spectrum (Fig. 4(b)) of Au-g-C_3_N_4_ confirmed the presence of an Au peak at ∼84 eV along with C and N, which verified the successful attachment of AuNPs onto the sheet-like structure of g-C_3_N_4_.^41^

The C 1s peaks were observed at 285 eV and 288.3 eV (Fig. 4(c)),^42^ which were assigned to the sp^2^-hybridized carbon atom and the carbon atom bonded to three nitrogen atoms –C(N_3_) of g-C_3_N_4_, respectively. The broad fitted peak of N 1s was observed at 398.5 eV (Fig. 4(d)),^43^ which were assigned to the nitrogen atom bonded to two carbon atoms (C–N–C) and the other small fitted peaks were attributed to nitrogen atoms bonded to the environment of three carbon atoms N–(C_3_) and to N–H bonding, respectively.^43^ The fitted spectrum of Au 4f (Fig. 4(e)) showed two peaks at 84.19 eV and 87.87 eV, which originated from the Au 4f_7/2_ and 4f_5/2_ electrons of the metallic behavior of gold.^41,44^ Therefore, the Au^3+^ ions were reduced to the Au^0^ oxidation state on the sheet like surface of g-C_3_N_4_.^45,46^Fig. 4(f) shows the combined C 1s spectrum of pure g-C_3_N_4_ and Au-g-C_3_N_4_ nanostructure. In case of AuNPs, the peak intensity is decreased (Fig. 4(f)) with the little shift in the binding energy. Therefore, its mainly related to a change of oxidation state of the element, here the shifting of binding energy relates to the changes of Au^3+^ to Au^0^ oxidation state. This analysis was supported by XRD, XPS, BET, and HR-TEM studies.

#### 3.1.5. Brunauer–Emmett–Teller, specific surface area analysis of the pure g-C_3_N_4_ and Au-g-C_3_N_4_ nanostructures

**Fig. 5:**
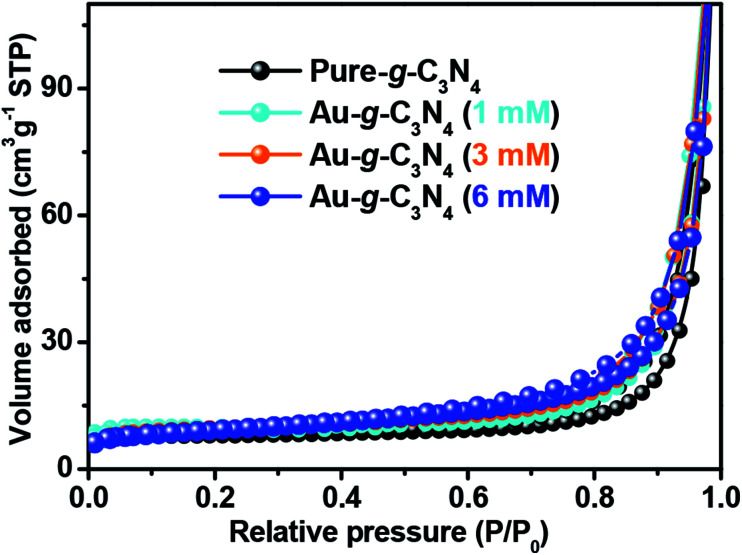
Nitrogen adsorption–desorption isotherm of the pure g-C_3_N_4_ and Au-g-C_3_N_4_ nanostructures.

N_2_-BET (Nitrogen Adsorption Brunauer–Emmett–Teller) was performed to detect the changes in the specific surface area of the as-fabricated samples. The measured specific surface areas of the pure g-C_3_N_4_ and Au-g-C_3_N_4_ nanostructures (1 mM, 3 mM, and 6 mM) were 31.0116 ± 0.3652 m^2^ g^−1^, 31.9655 ± 0.1336 m^2^ g^−1^, 34.9131 ± 0.3450 m^2^ g^−1^, and 41.1593 ± 0.4697 m^2^ g^−1^, respectively. In Fig. 5, the surface area of Au-g-C_3_N_4_ (6 mM) increased with increasing amount of precursor, which was much higher than that of pure g-C_3_N_4_. The higher specific surface area provides larger spaces to accommodate more charge storage and expose more active sites for the photochemical reaction. These results suggest that the visible light photoelectrochemical performance of the Au-g-C_3_N_4_ nanostructures could be improved greatly due to the higher specific surface area.

The nitrogen adsorption–desorption isotherm of pure g-C_3_N_4_ displays a hysteresis loop, suggesting the existence of mesopores.^42^ The AuNPs-loaded g-C_3_N_4_ exhibited much higher specific surface areas that of pure g-C_3_N_4_ (Table 1). The 6 mM AuNPs decorated g-C_3_N_4_ had a specific surface area of up to 41.15 m^2^ g^−1^. This shows that the optimal amount of AuNPs decorated g-C_3_N_4_ could provide more adsorption sites and photochemical reaction sites to improve the photoelectrochemical performance.

**Table 1:** Specific surface area measured from BET analysis of the pure-g-C__3__N__4__ and Au-g-C__3__N__4__ nanostructures

Sample name	BET surface area (m^2^ g^−1^)
Pure g-C_3_N_4_	31.0116 ± 0.3652
Au-g-C_3_N_4_ (1 mM)	31.9655 ± 0.1336
Au-g-C_3_N_4_ (3 mM)	34.9131 ± 0.3450
Au-g-C_3_N_4_ (6 mM)	41.1593 ± 0.4697

## 4. Photoelectrochemical studies

### 4.1. Photoelectrochemical studies of pure g-C_3_N_4_ and Au-g-C_3_N_4_ nanostructures using LSV, EIS and CV measurements

**Fig. 6:**
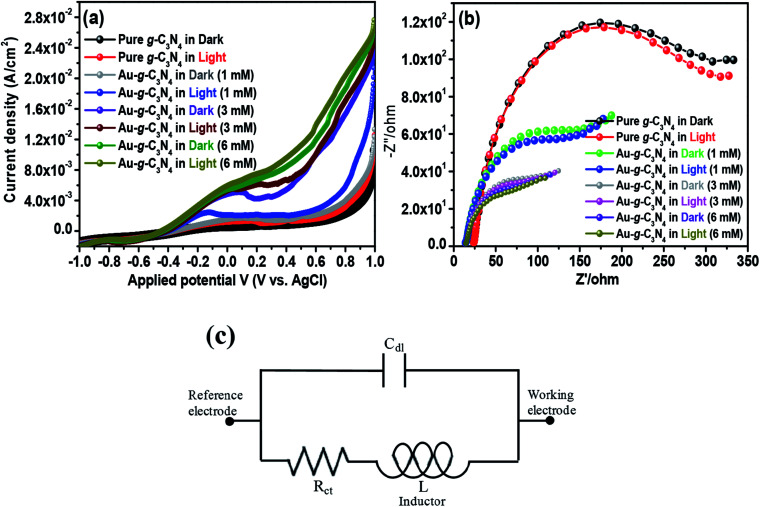
Visible light-induced photoelectrochemical performance (a) LSV, (b) EIS Nyquist plot of the pure g-C_3_N_4_ and Au-g-C_3_N_4_ as a photoelectrode in the dark and under visible light irradiation, and (c) equivalent circuit from the Nyquist plots.

Studies of the photoelectric behavior of pure g-C_3_N_4_ and Au-g-C_3_N_4_ nanostructures as a photoelectrode were performed using a standard three-electrode system. The measurements were taken under ambient conditions in the dark and under visible light irradiation in a 50 mL, a 0.2 M aqueous Na_2_SO_4_ solution as an electrolyte at room temperature. LSV and EIS were first performed in the dark and then under visible light irradiation (*λ* ≥ 400 nm) at a scan rate of 50 mV s^−1^ over the applied potential range, (−1 to 1 V).^41^ LSV is a voltammetry process, where the current at a working electrode is measured while the potential between the working electrode and reference electrode is swept linearly with time. LSV was performed in the dark and under visible light irradiation to provide evidence of the visible light-induced performance. The Au-g-C_3_N_4_ nanostructures (1–6 mM) displayed an enhanced photocurrent compared to pure g-C_3_N_4_ (Fig. 6(a)). The results in Fig. 6(a) showed that the photocurrent density depends basically on AuNPs deposition onto the sheet-like structure of g-C_3_N_4_. The current density increased significantly with increasing amount of AuNPs deposition. This higher increment in photocurrent density can be explained by the improved visible light absorption behavior of the material. The photocurrent depends largely on the number of photogenerated electrons; a higher number of electrons generated will improve the photocurrent density.^43,47^ The large number of electrons amassed in the conduction band of g-C_3_N_4_ resulted in a higher amount of photocurrent generation.^43,44,48^

The interfacial charge transfer rate is essential for improving the photoelectrode performance. Electrochemical impedance spectroscopy (EIS) was performed in the dark and under visible light irradiation to understand the charge separation process and transport properties of pure g-C_3_N_4_ and Au-g-C_3_N_4_ as a photoelectrode material, as shown in Fig. 6(b). In general, the complex impedance plot is normally presented as *Z*′/ohm *vs.* −*Z*′′/ohm, which initiates from the resistance and capacitance component of the electrochemical cell. A representative Nyquist plot includes one or more semicircular arcs with the diameter along the *Z*′/ohm axis.^41^ The semicircular arcs observed in the high and low frequency regions correspond to an electron transfer process, and its diameter represents the electron transfer or charge transfer resistance. In the present graph, a half circle arc with a reduced diameter for the Au-g-C_3_N_4_ was obtained compared to pure g-C_3_N_4_, which clearly reveals a rapid electron-transfer process in the case of the Au-g-C_3_N_4_ nanostructures under visible light irradiation. Generally, the small radius of the arc in the EIS spectra indicated lower electron transfer resistance at the surface of the photoelectrode, which is usually associated with the faster interfacial charge transfer. The concentration was increased from 1 mM to 6 mM under visible light irradiation; the EIS spectrum displayed a smaller arc radius of Au-g-C_3_N_4_. The performance of the as-fabricated nanostructure was better than that of pure g-C_3_N_4_.

Based on the EIS data (Fig. 6(b)), an equivalent circuit (Fig. 6(c)) fitted by the Zsimp Win 3.20d program with fine accuracy was obtained. Basically the equivalent circuit is used to analyze the measured impedance data. As shown in the circuitry, *R*_ct_ and *C*_dl_ represent the charge transfer resistance and double layer capacitance, and *L* describes the diffusion behavior at low frequencies, respectively. Table 2 shows the EIS fitting data obtained from the fitting of the equivalent circuits and the experimental values obtained from the impedance data. The fitting values of *R*_ct_ for Au-g-C_3_N_4_ nanostructures decrease from 1 mM to 6 mM. The higher concentration of AuNPs exhibit the small *R*_ct_ value which was much lower than that of pure-g-C_3_N_4_, which clearly suggested that the charge-transfer resistance is significantly reduced by anchoring of AuNPs onto sheet like structure of g-C_3_N_4_. The *C*_dl_ values displayed the opposite tendency as that of *R*_ct_ values. The low *R*_ct_ and high *C*_dl_ values indicate high electron transfer efficiency which further supports the improved photoelectrochemical performance of Au-g-C_3_N_4_ nanostructures.

**Table 2:** Fitting circuit values from equivalent circuit to analyze the Nyquist plots

Sample	*R*_ct_ (Ω cm^−2^)	*C*_dl_ × 10^8^ (F cm^−2^)
Pure-g-C_3_N_4_	21.82 ± 1.12	1.39 ± 0.14
Au-g-C_3_N_4_ (1 mM)	21.42 ± 0.96	2.01 ± 0.19
Au-g-C_3_N_4_ (3 mM)	19.26 ± 0.89	2.16 ± 0.21
Au-g-C_3_N_4_ (6 mM)	18.21 ± 1.00	2.49 ± 0.29

**Fig. 7:**
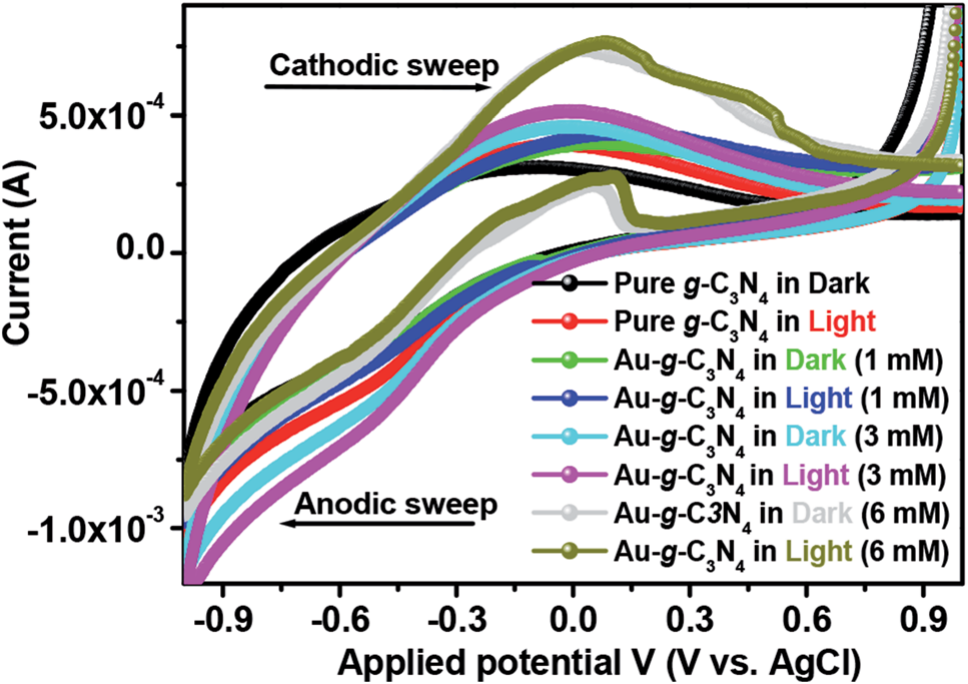
Cyclic voltammetry profile of pure g-C_3_N_4_ and Au-g-C_3_N_4_ nanostructures in a 0.2 M phosphate buffer (pH = 7) solution at 25 °C at a scan rate of 0.05 mV s^−1^.

The cyclic voltammogram (Fig. 7) of pure g-C_3_N_4_ and Au-g-C_3_N_4_ nanostructures were obtained in the dark and under visible light irradiation at a scan rate of 0.05 mV s^−1^. The CV plot of the Au-g-C_3_N_4_ nanostructures showed an improved positive and negative sweep, indicating their pseudo capacitive nature. The peak current of the Au-g-C_3_N_4_ nanostructures from 1 mM to 6 mM increased linearly in the dark and under visible light irradiation with a positive shift of the cathodic peak and a negative shift of the anodic peak.^41,45^ The improved anodic and cathodic peak verified the amended current transfer ability of the Au-g-C_3_N_4_ nanostructures under visible light irradiation, which also reveals better capacitive performance of the as-fabricated nanostructures. Consequently, the improved capacitive performance of the Au-g-C_3_N_4_ nanostructures can be attributed to its improved charge loading ability and the synergistic effect of AuNPs and g-C_3_N_4_ under visible light irradiation.^46,49,50^

### 4.2. Incident photon-to-current conversion efficiency (IPCE) test

**Fig. 8:**
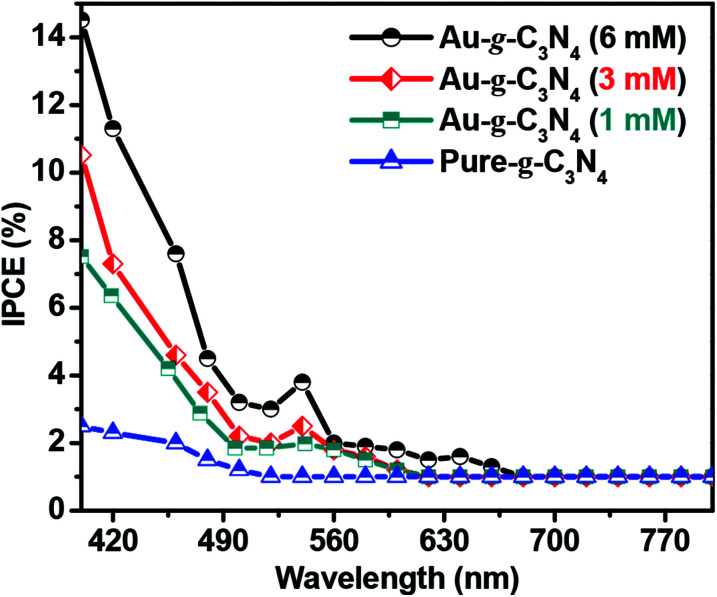
IPCE graph for pure-g-C_3_N_4_ and Au-g-C_3_N_4_ (1–6 mM) nanostructures at 1.2 eV *vs.* Ag/AgCl in 0.2 M Na_2_SO_4_.

To investigate the photoresponse of pure-g-C_3_N_4_ and Au-g-C_3_N_4_ (1–6 mM) nanostructures, IPCE measurements at 1.2 eV *vs.* Ag/AgCl as the reference electrode are presented in Fig. 8. The IPCE can be expressed as follows:^51,52^2
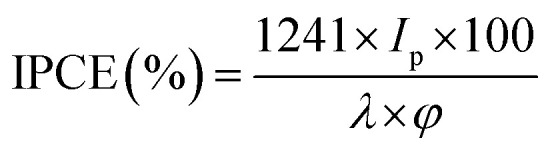
where *λ*, *φ* and *I*_p_ denote the wavelength of the incident light (nm), the irradiation power (mW cm^−2^), and the photocurrent density (A cm^−2^) measured at the corresponding wavelength, respectively.

Fig. 8 of IPCE tests shows a visible light response in case of higher Au-g-C_3_N_4_ nanostructures. The absorption threshold of g-C_3_N_4_ is approximately 460 nm, with a lower IPCE value. Anchoring of AuNPs onto sheet like structure of g-C_3_N_4_ results in substantial enhancement of the IPCE values for Au-g-C_3_N_4_ nanostructures (1–6 mM) as follows: 14.6%, 10.5%, and 7.4% under visible light irradiation respectively. In addition, the small hump appeared in the visible region which is caused primarily by the SPR effect of AuNPs. While in the case of bare g-C_3_N_4_, the IPCE performance was very less (2.2%) without any hump in visible region as compared to Au-g-C_3_N_4_ which further confirms the role of AuNPs with spatial effect of SPR. This result indicates the anchored AuNPs shows SPR effect which helps to improve the photoelectrochemical performance of nanostructures.

The progressive visible light-induced photoelectrochemical performance using Au-g-C_3_N_4_ nanostructures confirmed the successful anchoring of AuNPs onto the sheet-like structure of g-C_3_N_4_. The improved photocurrent performance revealed the interfacial interaction and charge transfer between the AuNPs and g-C_3_N_4_, which could explain the enhanced photoelectrochemical performance of the Au-g-C_3_N_4_ nanostructures.

## 5. Proposed electron transfer mechanism of Au-g-C_3_N_4_ nanostructures under visible-light irradiation

Generally, in case of a semiconducting material, visible-light irradiation plays a significant role in the excitation of electrons from the valence band (VB) to the conduction band (CB). In presence of visible-light irradiation (*λ* ≥ 400 nm) g-C_3_N_4_ nanostructures excited and electrons (e^−^) from the VB transfer to the CB, leaving the holes (h^+^) in the VB, thereby forming the electron–hole pairs.^21,53–57^ The photogenerated electrons can rapidly transfer the AuNPs due to their intimate interfacial contact between g-C_3_N_4_ and AuNPs, resulting in a significantly improved lifetime of the photogenerated electron–hole charge carrier.^41,56^

**Fig. 9:**
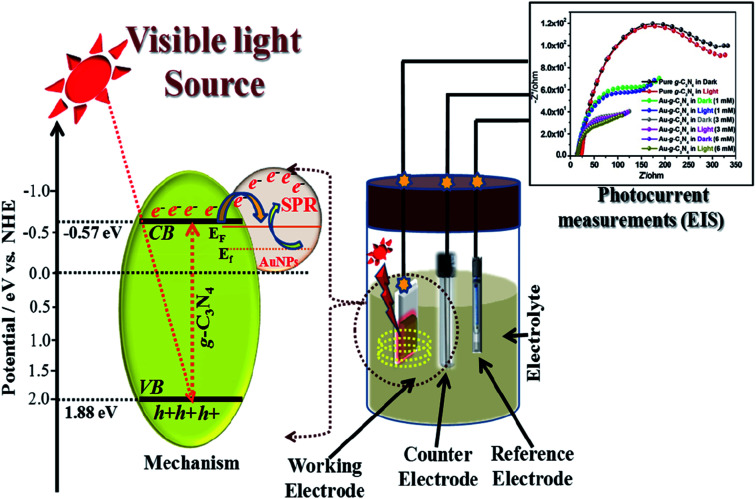
Proposed schematic charge transfer mechanism for the photocurrent performance over the visible light-induced Au-g-C_3_N_4_ nanostructures as a photoelectrode.

Fig. 9 shows a schematic diagram of the probable procedure for the charge separation in Au-g-C_3_N_4_nanostructures under visible-light irradiation. As shown in Fig. 9 visible-light irradiation was focused on the as-prepared electrode on FTO glass, which was dipped in the electrolyte solution. The electrolyte solution acts as a donor or acceptor to contribute or receive electrons from the electrodes. The Au-g-C_3_N_4_ nanostructures sample showed higher photocurrent performance (1–6 mM) because of its tuned optical properties compared to the bare g-C_3_N_4_. The counter and reference electrode measure the photocurrent with the help of the electrolyte solution and finally we recorded the improved performance of photocurrent form Au-g-C_3_N_4_ nanostructures.

## 6. Conclusions

This paper reported a facile, green, and competent approach for the fabrication of Au-g-C_3_N_4_nanostructures with spherical and uniform sized AuNPs with a high surface area (41.1593 m^2^ g^−1^) and improved photoelectrochemical performance. A single strain developed biofilm was used as a tool to reduce Au^3+^ to Au^0^ and Au-g-C_3_N_4_ nanostructures (1 mM, 3 mM and 6 mM) were fabricated. The anchoring of AuNPs onto the sheet-like structure of g-C_3_N_4_ produced promising photoelectrode material for real photonic devices. The boosted photoelectrochemical performance of Au-g-C_3_N_4_ nanostructures compared to that of pure g-C_3_N_4_ were explained based on the strong visible-light absorption, superior photocurrent generation, surface plasmon effect of AuNPs, and lower photoluminescence intensity. The spherical shape, size and uniform dispersion of the AuNPs over the g-C_3_N_4_ sheet were valuable for increasing the photocurrent performance. These findings were attributed mainly to the higher visible-light absorption by AuNPs ensuring the formation of a large number of photogenerated electron–hole pairs. This large number of exciton was transferred immediately through the polar-semiconductor–noble-metal interface to the-sheet like structure of g-C_3_N_4_, which inhibited the charge recombination process and increased the photocurrent performance.

## Conflicts of interest

The authors declare no competing financial interests.

## Supplementary Material

RA-008-C8RA00690C-s001
