# Supplementary material for: Environmentally sustainable biogenic fabrication of AuNP decorated-graphitic g-C3N4 nanostructures towards improved photoelectrochemical performances
Source: RSC Adv. 2018 Apr 16;8(25):13898–909. doi: 10.1039/c8ra00690c (PMC9079820; doi:10.1039/c8ra00690c)
Supplement: RA-008-C8RA00690C-s001 [file RA-008-C8RA00690C-s001.pdf]

Electronic Supplementary Material (ESI) for RSC Advances.
This journal is © The Royal Society of Chemistry 2018

Electronic Supplementary Information

Environmentally Sustainable Biogenic Fabrication of AuNPs decorated-graphitic *g*-C_3N_4 Nanostructures towards Improved Photoelectrochemical Performances

Mohammad Ehtisham Khan[1], Mohammad Mansoob Khan[2*], Moo Hwan Cho[1*]

[1]*School of Chemical Engineering, Yeungnam University, Gyeongsan, Gyeongbuk 38541, South Korea. Phone: +82-53-810-2517, Fax: +82-53- 810-4631.*
[2]*Chemical Sciences, Faculty of Science, Universiti Brunei Darussalam, Jalan Tungku Link, Gadong, BE1410, Brunei Darussalam.*

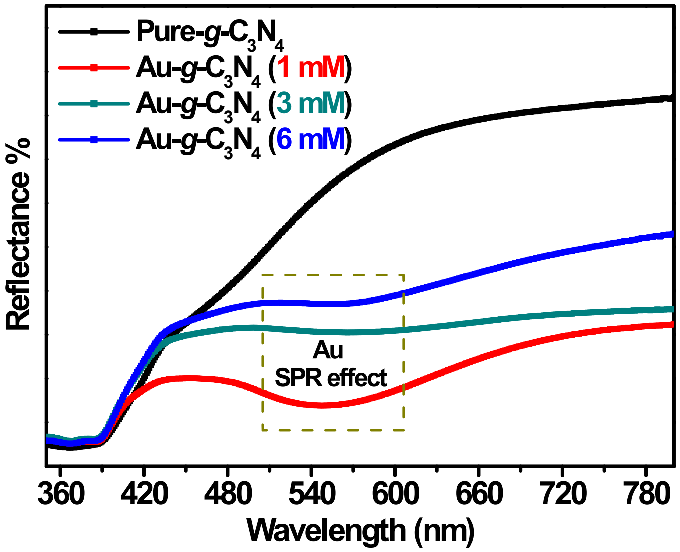

Fig. S1 Reflectance spectra of pure *g*-C_3N_4 and Au-*g*-C_3N_4 nanostructures.

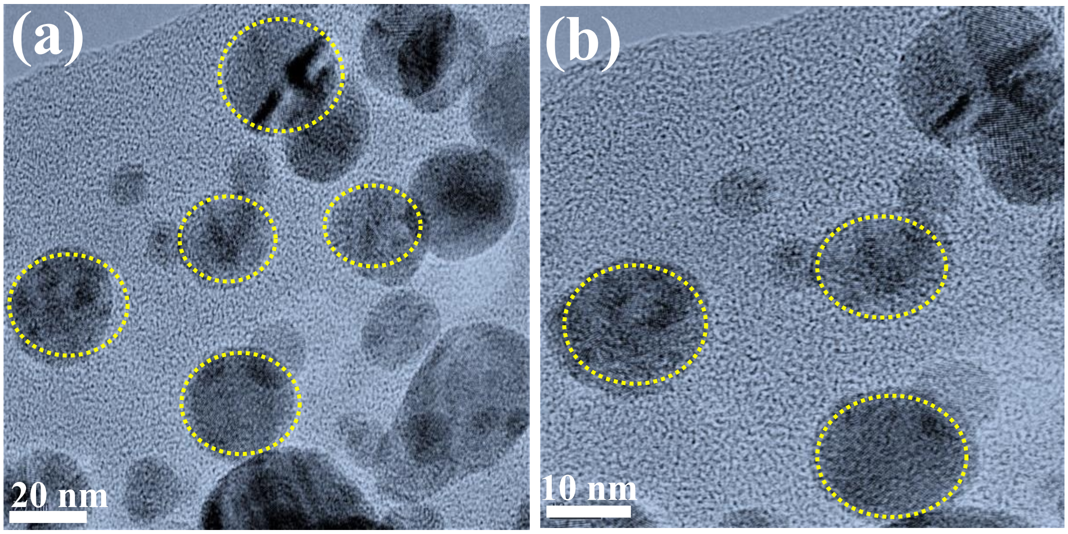

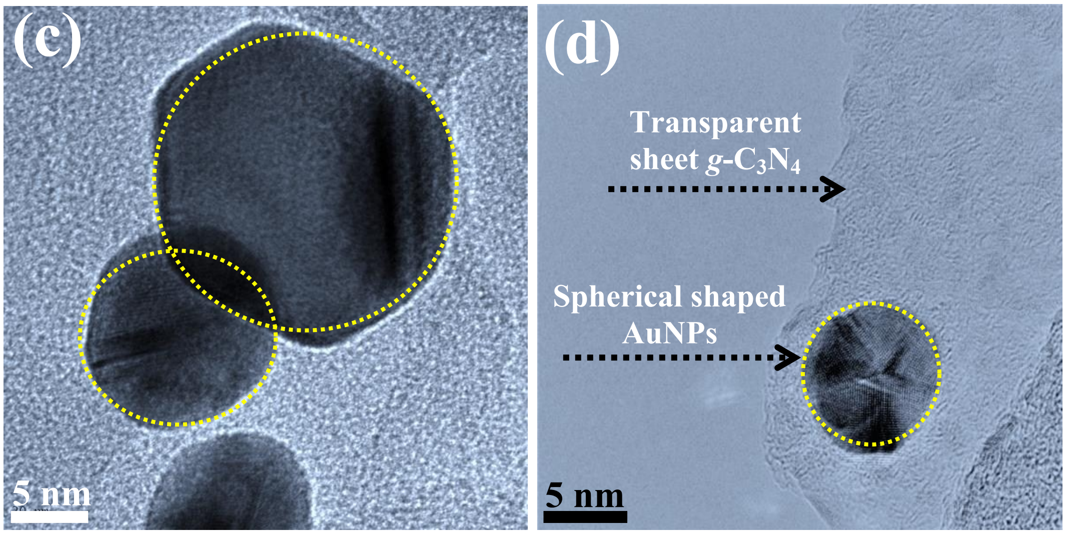

Fig. S2 HR-TEM images of Au-*g*-C_3N_4 nanostructures.

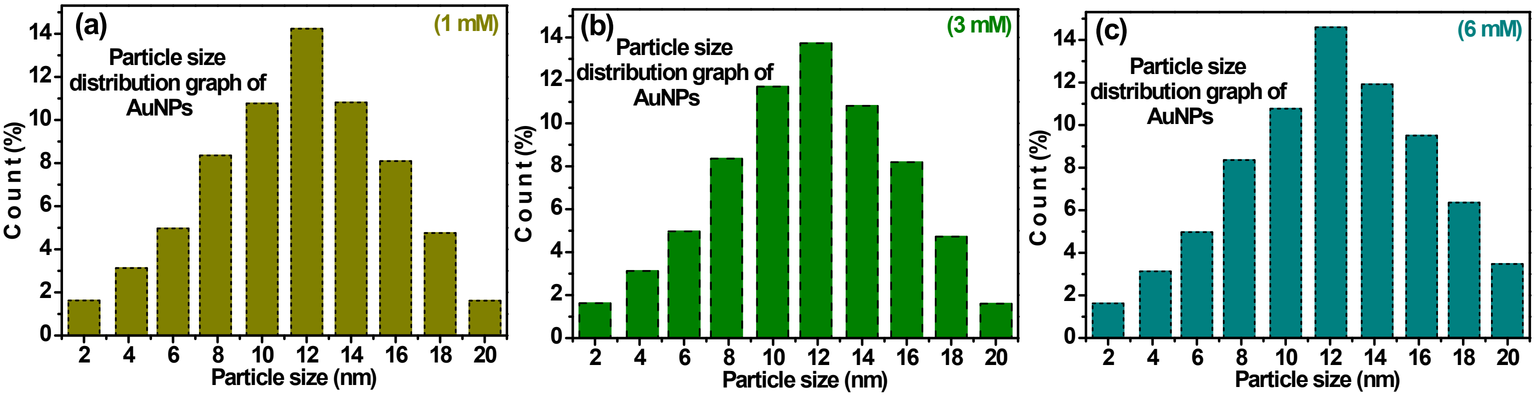

Fig. S3 (a, b and c) Particle size distribution graph of Au-*g*-C_3N_4 nanostructures.
